# Proline Metabolism Process and Antioxidant Potential of *Lycium ruthenicum* Murr. in Response to NaCl Treatments

**DOI:** 10.3390/ijms241813794

**Published:** 2023-09-07

**Authors:** Richard John Tiika, Huirong Duan, Hongshan Yang, Guangxin Cui, Fuping Tian, Yongtao He, Yanjun Ma, Yi Li

**Affiliations:** 1 College of Forestry, Gansu Agricultural University, Lanzhou 730070, China; 2 Lanzhou Institute of Husbandry and Pharmaceutical Science, Chinese Academy of Agricultural Sciences, Lanzhou 730050, China

**Keywords:** *Lycium ruthenicum* Murr., NaCl treatments, proline, metabolic enzymes, antioxidants, gene expression

## Abstract

Salinity influences the level of antioxidants and proline content, which are both involved in the regulation of stress responses in plants. To examine the interplay between the antioxidant system and proline metabolism in plant stress acclimation, explants of *Lycium ruthenicum* were subjected to NaCl treatments, and the growth characteristics, antioxidant enzyme activities, proline accumulation, and metabolic enzyme content were analyzed. The results revealed that NaCl concentrations between 50 to 150 mM have a positive effect on the growth of *L. ruthenicum* explants. Increasing NaCl concentrations elevated the activities of superoxide dismutase (SOD) and catalase (CAT), while hydrogen peroxide (H_2_O_2_) content was inhibited, suggesting that the elevated antioxidants play a central protective role in superoxide anion (O_2_^•−^) and H_2_O_2_ scavenging processes in response to NaCl treatments. Also, high proline levels also protect antioxidant enzyme machinery, thus protecting the plants from oxidative damage and enhancing osmotic adjustment. Increasing levels of pyrroline-5-carboxylate synthetase (P5CS), pyrroline-5-carboxylate reductase (P5CR), and ornithine-δ-aminotransferase (δ-OAT) were observed, resulting in elevated level of proline. In addition, the expression levels of *LrP5CS1*, *-2*, *-3*, *LrOAT-1*, and *-2* were promoted in NaCl treatments. According to the combined analysis of metabolic enzyme activities and their relative expression, it is confirmed that the glutamate (Glu) pathway is activated in *L. ruthenicum* faced with different levels of NaCl concentrations. However, Glu supplied by δ-OAT is fed back into the main pathway for proline metabolism.

## 1. Introduction

It has been shown that the key abiotic factor limiting plant growth is soil salinity, which negatively affects a number of biochemical and physiological processes. In order to preserve vital cellular functions such as photosynthesis, antioxidant synthesis, mineral homeostasis, osmolyte synthesis, and respiration from salt stress, plants modify their metabolic mechanisms [1,2]. These mechanisms work together by altering the metabolism and activating biochemical pathways. They can also affect the production of chemical substances that were absent or present in low levels before the onset of stress [3,4].

Salt stress in plants leads to oxidative stress and an increase in the production of reactive oxygen species (ROS), which are regulated by the antioxidant system [2,5]. To protect delicate cell membranes from ROS-induced damage, plants have developed sophisticated ROS scavenging systems; these systems include enzymatic and nonenzymatic antioxidant activities. Enzymatic antioxidants such as superoxide dismutase (SOD) are believed to accelerate the reaction of superoxide anion (O_2_^•−^) with itself to form hydrogen peroxide (H_2_O_2_) in chloroplasts and mitochondria, while catalase (CAT) and peroxidase (POD) can eliminate H_2_O_2_ and scavenge superoxide anion (O_2_^•−^) [6,7]. SOD, CAT, POD, and other enzymes associated with the ascorbate–glutathione pathway are crucial to the scavenging of H_2_O_2_. The accumulation of H_2_O_2_, which results from the canalization reaction of the SOD enzyme, is prevented by the ascorbate–glutathione cycle [8]. The hydroxyl radical (·OH), which is very reactive and the most toxic oxide, can react with all macromolecules without discrimination. Although the specific scavengers of HO have not been fully elucidated, it is believed that SOD and CAT can reduce or prevent its formation by combining their actions [9]. CAT is a tetrameric heme that catalyzes the conversion of H_2_O_2_ formed during the β-oxidation of fatty acids to water and molecular oxygen [10]. POD and CAT appear to have a critical protective function in the scavenging mechanism when regulated with activity of SOD [10,11].

Proline is one of the numerous osmolytes, and among them is the most frequently accumulated in stressed plants [12]. It is now widely acknowledged that proline has multiple functions, as much attention has been paid to its function and metabolism in plants under stress. Aside from its osmotic function, proline is also believed to serve other functions in stress situations, such as carbon and nitrogen energy storage, scavenging H_2_O_2_, buffering cytosolic pH and redox adjustment status of cells, stabilizing protein structure, and finally acting as a transmitter for stress tolerance [13,14,15]. A significant amount of research suggests that exogenously administered proline could improve stress resistance in plants. For example, numerous plant species that grew under salt stress benefited from osmoprotection and growth-promoting effects after exogenous application of proline [16]. Also, exogenous administration of proline increased the activity of many antioxidant enzymes that protect cell membranes from salt-induced oxidative damage [17].

In plants, the biosynthesis of proline consists of two sequential pathways: the glutamate (Glu) and ornithine (Orn) pathways [18,19]. Firstly, pyrroline-5-carboxylate synthetase (P5CS) reduces Glu in the cytosol and chloroplasts to glutamate semialdehyde (GSA), where it is then rapidly converted into pyrroline-5-carboxylate (P5C), the common intermediate for both proline and ornithine metabolism. P5C is subsequently reduced to proline by P5C reductase (P5CR) [20]. Secondly, the Orn pathway occurs in mitochondria where Orn is transaminated to P5C by the enzyme ornithine-δ-aminotransferase (δ-OAT), and P5C is subsequently transferred to the cytosol and converted to proline by the enzyme P5CR. In mitochondria, proline is degraded by successive activities of proline dehydrogenase (ProDH) and pyrroline-5-carboxylate dehydrogenase (P5CDH), which generate P5C and Glu, respectively. Among the aforementioned enzymes necessary for proline metabolism, P5CS is typically regarded as the primary enzyme for proline synthesis, whereas ProDH is crucial for degradation. In *Arabidopsis thaliana*, the Orn pathway has been shown to be more important for arginine catabolism and not for proline generation [20,21]. However, in some plants, the Orn pathway may be critical for proline synthesis under various abiotic stresses [22,23].

*Lycium ruthenicum* Murr. (Solanaceae), commonly known as wolfberry, is a member of xerohalophytes that has developed strong adaptability to saline conditions and is widely distributed in Asia, Europe, and Africa’s arid and semiarid regions [24]. It is an excellent shrub that is crucial for the restoration of desert ecosystems and improving soil salinity/alkalinity, which is necessary for ecosystem protection due to its special physiological traits in terms of both salt and drought tolerance [25,26,27]. This shrub has been introduced in China and suggested as a possible crop for sustainable saline agriculture; also, because of its medicinal, economic contribution and resistance to saline soils, it has attracted widespread attention in some parts of the world [28,29,30,31]. Previous scientific works on *L. ruthenicum* have predominantly concentrated on ecological adaptation, anthocyanin content, economic, and medicinal benefits [32,33]. Also, earlier studies of salt stress in *L. ruthenicum* showed a drastic increase in proline and antioxidant contents [32,34]; however, the specific mechanisms of proline metabolism and antioxidant enzyme activities have not been reported until now. Therefore, we investigated the salt-induced mechanism of proline metabolism and SOD, CAT, and POD activities in *L. ruthenicum*. It has been vividly reported that proline accumulation in plants is a combined process of catabolism, biosynthesis, and transport [12]; therefore, this prompted us to identify proline biosynthesis- and catabolism-related genes from *L. ruthenicum*, investigate their expression profiles and the activities of enzymes involved in the steps of proline metabolism, and further confirm the proline metabolism pathway under NaCl treatments in *L. ruthenicum*.

## 2. Results

### 2.1. Growth and Structural Responses of Leaf Surface to Different NaCl Treatments

To explore phenotypic traits and biomass response to NaCl treatments, shoot height, root length, leaf fresh weight, stem fresh weight, and root fresh weight were measured. We found differences in biomass and morphological traits, although no visible damage was observed. Compared to the control, explants treated with different NaCl concentrations showed phenotypic changes in shoot height and root length and also resulted in changes in fresh biomass (Table 1). Significant (*p* ≤ 0.05) trends were observed in shoot length, root length and root fresh weight of explants treated with 100 mM NaCl, which were 1.03, 1.04, and 0.34 times greater than the control, respectively. At high concentration (300 mM NaCl), the size of explants decreased compared with the control. NaCl treatments also resulted in changes in biomass, for instance, fresh weight of leaves and stems was significantly increased when treated with 150 mM NaCl, while the fresh weight of roots was higher when treated with 100 mM NaCl compared to control explants. The highest decrease in biomass occurred in explants treated with 300 mM NaCl. These changes are used as indicators of growth inhibition by NaCl treatment. These findings were confirmed in our study, in which 50 and 100 mM promoted root length; 100 mM promoted shoot length, root length, and root fresh weight; while 150 induced leaf and stem fresh weight.

**Figure 1:**
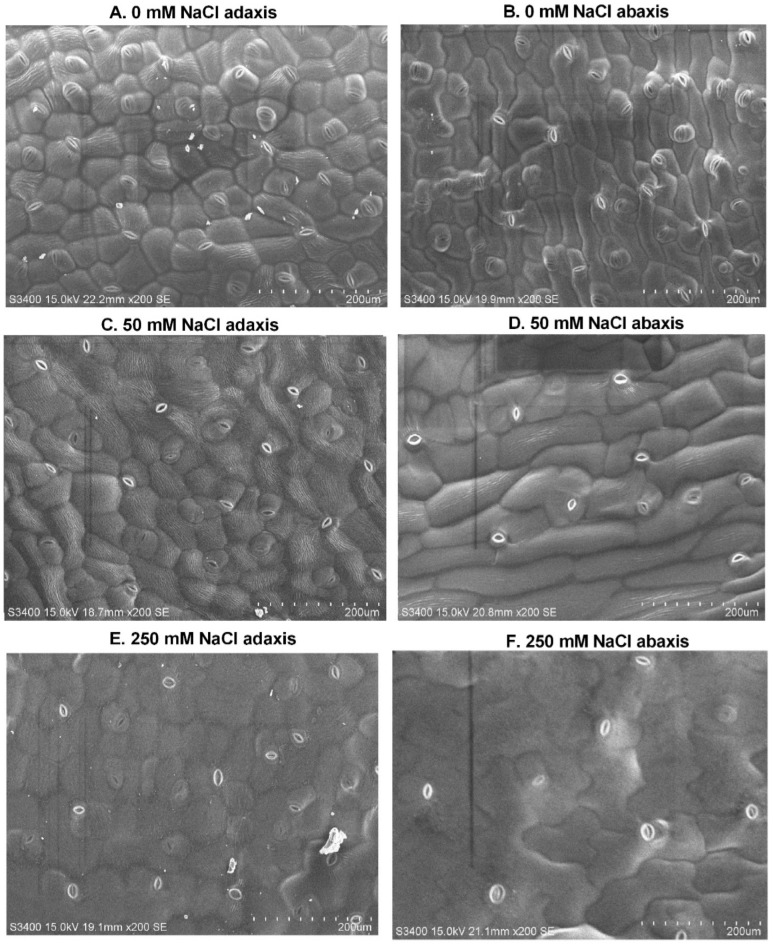
Leaf surface morphology under different levels of NaCl stress in *L. ruthenicum.* (**A**,**B**) 0 mM for adaxial and abaxial surfaces, (**C**,**D**) 50 mM for adaxial and abaxial surfaces, (**E**,**F**) 250 mM for adaxial and abaxial surfaces.

In addition, the observed leaves of *L. ruthenicum* were amphistomatic, with stomata on both the adaxial and abaxial surfaces. Our results revealed that the size of stomata was significantly smaller on both leaf surfaces in the control and high NaCl treatment than in moderate (50 mM) NaCl. The stomata density decreased with increasing NaCl, and the adaxial leaf surface had highest number of stomata than abaxial surface. The size of guard cells was higher in 250 mM NaCl treatments on the adaxial surface but higher on the abaxial surface in control. With increasing NaCl concentration, the size of leaf epidermal cells increased by 46.7% and 58.6% on both adaxial and abaxial surfaces, respectively, compared with the control (Table 2 and Figure 1).

### 2.2. Responses of Proline, Soluble Protein, and Leaf Osmotic Potential (Ψs) to Different Concentrations of NaCl

**Figure 2:**
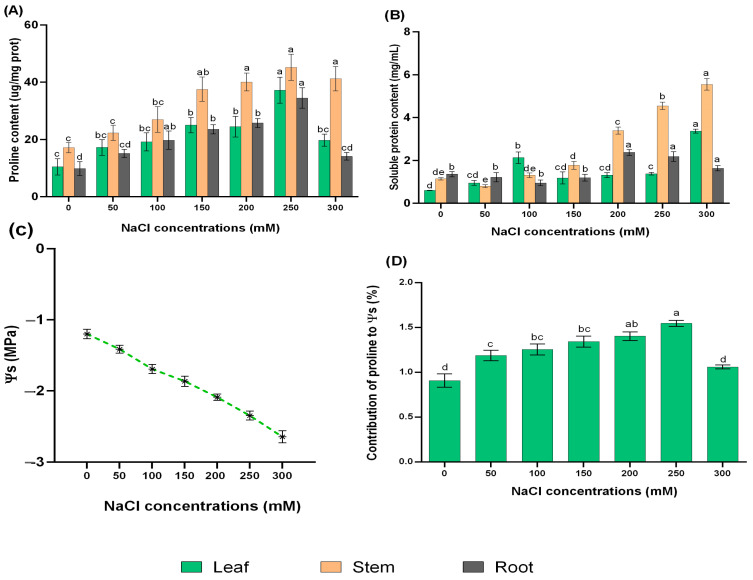
Effects of NaCl treatments on (**A**) proline content, (**B**) soluble protein content, and (**C**) osmotic potential in leaves. (**D**) Contribution of proline to osmotic potential (%) in leaves. Different letters on the bars indicate significant differences of tissues among different NaCl treatments (*p ≤* 0.05).

Under adverse conditions, plants achieve normal metabolic activities by balancing osmoprotectants in their system and developing strategies suitable for survival in their habitat. To assess whether NaCl treatments can increase osmotic adjustment of *L. ruthenicum* explants, soluble protein, osmotic potential, and proline content were measured. The proline content gradually increased with the increase in external NaCl concentrations, and the highest value was detected under 250 mM NaCl, which was 2.58-, 2.64-, and 2.51-fold higher than that of control explants in leaf, stem, and root, respectively. When treated with 300 mM NaCl, there was no evidence of damage to the explants, although the proline content decreased (Figure 2A).

In addition, the soluble protein concentration in the leaf and stem of *L. ruthenicum* explants was greatly increased under 300 mM NaCl, by 4.85- and 5.55-fold, respectively, compared to the control. The root also showed a discrete increase, with the maximum content recorded under 200 mM NaCl treatment (Figure 2B).

Ψs in leaves of *L. ruthenicum* explants was very sensitive to high NaCl treatment and gradually decreased with increasing NaCl concentrations. The greatest reduction in Ψs occurred in explants treated with a higher NaCl concentration when it dropped to −2.65 MPa compared with control explants (Figure 2C). Moreover, the contribution of leaves proline to Ψs increased to 1.5% under 250 mM NaCl (Figure 2D).

### 2.3. The Response of Antioxidant Enzymes and H_2_O_2_ to NaCl Treatments

**Figure 3:**
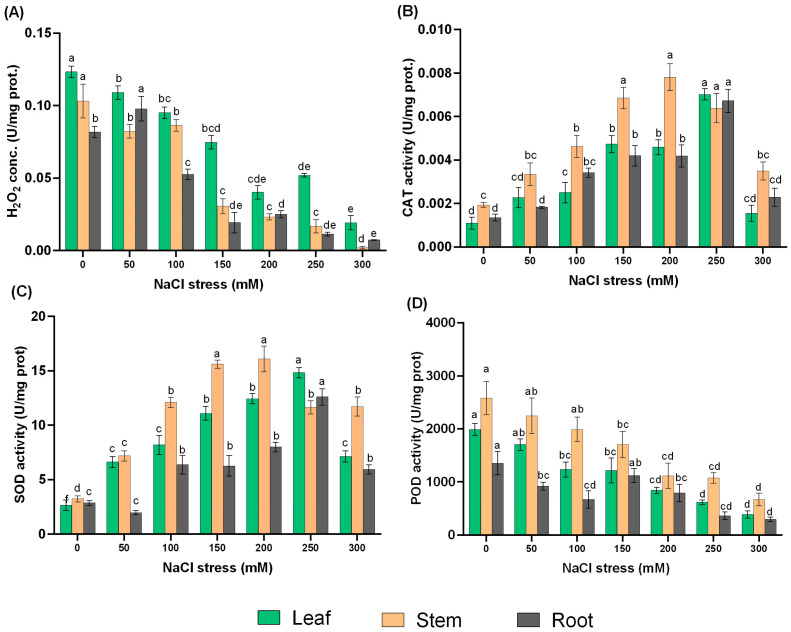
H_2_O_2_ content and antioxidant enzymes in *L. ruthenicum* under NaCl treatments. (**A**) H_2_O_2_ content, (**B**) CAT activity, (**C**) SOD activity, and (**D**) POD activity. Different letters on the bars show significant differences of tissues among different NaCl treatments (*p ≤* 0.05).

H_2_O_2_ content and antioxidant enzyme activities are protective mechanisms against salt stress, and evaluation of their activities may contribute to a deeper understanding of the adaptation response of *L. ruthenicum* to salinity. Therefore, we analyzed H_2_O_2_ content and activities of three antioxidant enzymes (CAT, SOD, and POD). The H_2_O_2_ content showed a decreasing trend as a function of NaCl treatments, with the highest content in leaf and stem under control, whereas 50 mM NaCl treatment provided the highest concentration in the root compared with other NaCl treatments (Figure 3A).

In this study, the activities of SOD and CAT revealed a sharp increase with increasing NaCl treatment and then peaked at 200 mM treatment in the stem and 250 mM treatment in the leaf and root when each concentration gradient was compared with the control (Figure 3B,C).

Interestingly, the activity of POD decreased in almost all NaCl treatments and in all tissues compared to the control. Though the activity of POD decreased, the stem revealed higher activity among all the NaCl treatments, followed by the leaf (Figure 3D).

### 2.4. Effect of NaCl Treatments on the Activities of Proline Metabolic Enzymes

**Figure 4:**
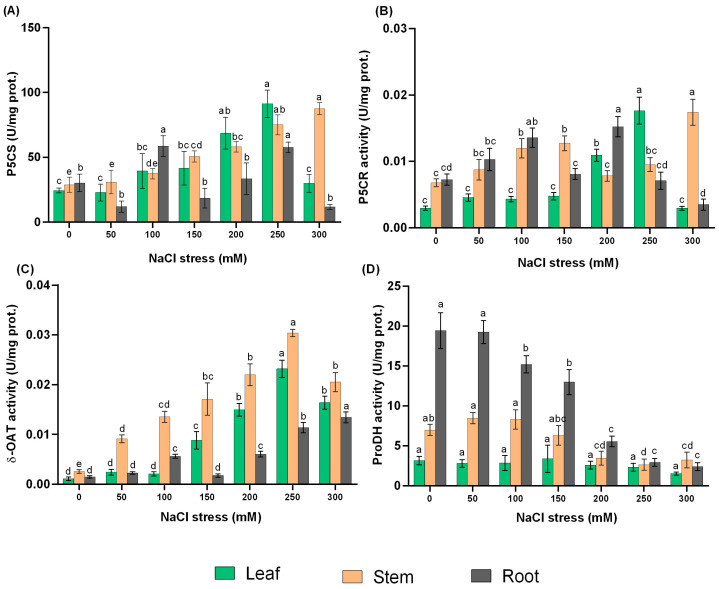
Changes in metabolism enzyme contents of *L. ruthenicum* explants under different NaCl treatments. (**A**) P5CS activity, (**B**) P5CR activity, (**C**) δ-OAT activity, and (**D**) ProDH activity. Different letters on the bars indicate significant differences of tissues among different NaCl treatments (*p* ≤ 0.05).

The results in Figure 4A show that as the concentration of NaCl treatments increased, the activity of the key bifunctional enzyme P5CS increased significantly (*p* ≤ 0.05). Among the different NaCl concentrations, 250 and 300 mM exhibited the highest P5CS activity in leaf and stem, with 3.74- and 3.04-fold increases, respectively, compared with the control. 

Under NaCl treatments, P5CR activity increased significantly (*p* ≤ 0.05) at 200, 250, and 300 mM NaCl for leaf, stem, and root, respectively, by 2.10-, 5.97-, and 2.56-fold compared to the control treatment (Figure 4B).

It is observed that high NaCl treatments can significantly (*p* ≤ 0.05) increase δ-OAT activity in *L. ruthenicum* explants, with the highest increase under 250 mM treatment. The root initially increased, then decreased at 150 mM treatment and increased again to optimum at 300 mM treatment with a 9.17-fold increase compared with control (Figure 4C).

Leaf proDH activity remained stable with increasing of NaCl concentration, while the stem activity slowly decreased with increasing NaCl concentration. With increasing NaCl levels, the activity of ProDH in the root decreased sharply after treatment with 50 mM NaCl (Figure 4D).

### 2.5. Bioinformatic Analysis of the Proline Metabolic-Related Genes

After searching 43,573 unigenes for all possible metabolic enzyme genes from transcriptome data of *L. ruthenicum*. Only candidates with fragments per kilobase of transcripts per million mapped reads (FPKM) values ≥ 5 were selected for further analysis under NaCl treatments (Supplementary data file S1). The candidate genes were renamed using the overlapping prefix “*Lr*” for *L. ruthenicum*; *LrP5CS1*, *-2* and *-3*, *LrP5CR*, *LrOAT1* and *-2*, *LrProDH1* and *-2*, and *LrP5CDH*, respectively.

Blast analysis revealed that these identified genes have high similarity to known genes in the GenBank. The deduced amino acid sequences of LrP5CS1, -2, and -3 were 98% identical to *Capsicum annuum* (KAF3612993.1), *Lycium chinense* (AIS36041.1), and *Capsicum chinense* (PHU13933.1) in the GenBank. The LrP5CR candidate protein also showed very high identity with P5CRs from other plants and had the highest identity of 95% with P5CR in *Solanum stenotomum* (XP_049384604.1). Both LrOAT1 and -2 candidate proteins also had a high identity of 95% with OAT in *C. chinense* (PHU09018.1) in the GenBank. The putative LrPDH1 and -2 proteins were 89% and 83% identical to *Solanum verrucosum* (XP_049346113.1) and *Capsicum baccatum* (PHT57387.1), respectively. The deduced protein sequences were further characterized by their basic features such as the number of amino acid residues, molecular weight, prediction of subcellular localization, and isoelectric point (pI) (Table 3).

### 2.6. Differential Expression Analysis of the Proline Metabolism-Related Genes

**Figure 5:**
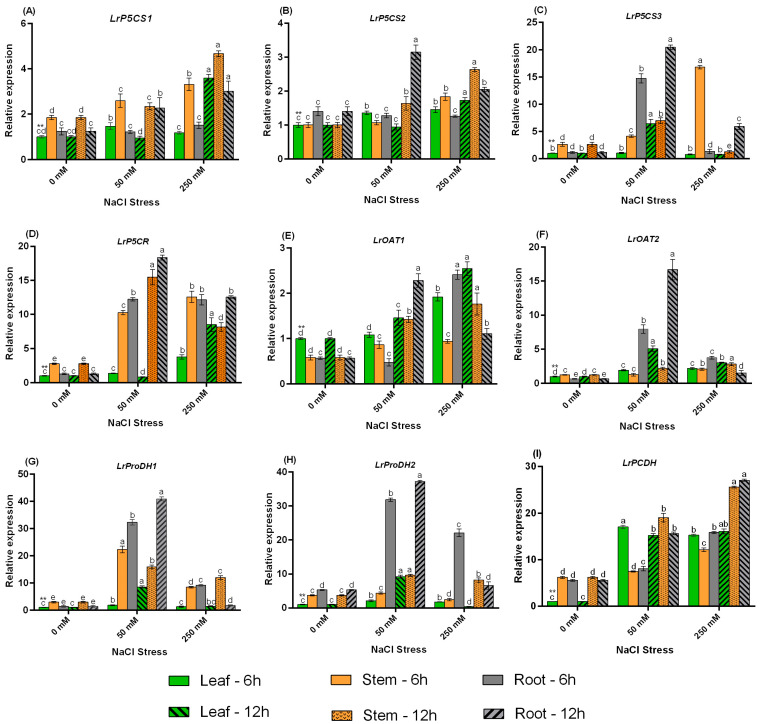
The relative expression patterns analysis of proline metabolism genes in *L. ruthenicum* under NaCl treatment. The name of the gene is at the top of each bar graph; (**A**) *LrP5CS1*, (**B**) *LrP5CS2*, (**C**) *LrP5CS3*, (**D**) *LrP5CR*, (**E**) *LrOAT1*, (**F**) *LrOAT2*, *(***G**) *LrProDH1*, (**H**) *LrProDH2*, and (**I**) *LrP5CDH*. Different letters on the bars indicate significant differences between NaCl treatments in each tissue and time (*p* ≤ 0.05) and the asterisks (**) represent reference condition for gene expression.

The expression levels of all the proline metabolism enzyme genes were analyzed in a variety of tissues under 50 and 250 mM NaCl treatments. All the three *P5CS* genes are expressed at different times in different tissues depending on NaCl concentration. The relative expression level of *LrP5CS1* was significantly expressed in almost all tissues under 50 and 250 mM NaCl treatments. The relative expression of *P5CS1* was much higher in all tissues under 250 mM treatment, especially with increasing treatment time; the relative expression under 50 mM treatment was also higher in leaf and stem after 6 h, and in stem and root after 12 h compared with control. *LrP5CS2* is also strongly regulated in almost all tissues under both NaCl concentrations, although there are differences in expression patterns at different concentrations and treatment durations. Relative expression peaked in root and stem under 50 mM NaCl at 12 h compared with control but expressed higher in leaf at 6 h. Expression in the root peaked after 12 h at 50 mM treatment but decreased significantly at 250 mM treatment; an increasing trend in expression was observed in the leaf and stem. *LrP5CS3* showed a higher expression pattern at both NaCl concentrations, except in the leaf under 250 mM treatment, which was not expressed throughout the treatment periods. In general, the expression of *P5CS3* under 50 mM is higher than under 250 mM, except for 250 mM treatment under 6 h leaf (Figure 5A–C).

The expression analysis revealed that *LrP5CR* was significantly expressed in almost all tissues under the NaCl treatments compared with control. The expression patterns of stem and root increased and peaked under 50 mM treatment at 12 h by 16.6- and 19.1-fold, respectively, but decreased significantly at 250 mM (*p ≤* 0.05); the expression of leaf was significantly higher under 250 mM treatment than under control and 50 mM treatments. In 250 mM treatment, expression in the stem peaked after 6 h and was 11.6-fold higher than the control (Figure 5D).

The expression pattern of *LrOAT1* in leaf and stem increased in a concentration-dependent manner, peaking at 250 mM treatment compared with control and 50 mM treatments. The expression of *LrOAT2* increased under 50 mM and decreased under 250 mM in leaf and root but increased in stem and peaked under 250 mM NaCl with longer treatment duration. The expression of both genes in the root was higher at 250 mM 6 h after treatment (Figure 5E,F).

*LrProDH* genes are also affected by NaCl stimulation, with different expression patterns under 50 and 250 mM treatments. With the exception of *LrProDH1* in the stem, the expression patterns of both genes (*LrProDH1* and *-2*) increased in the leaf, stem, and root under 50 mM treatment and peaked at 12 h compared with the control. Expression of *LrProDH1* peaked only in the root, whereas *LrProDH2* peaked in the leaf and root at 6 h under 250 mM treatment. Also, under 50 mM at 12h, the expression levels are much higher compared to 250 mM NaCl treatment (Figure 5G,H).

Finally, NaCl treatments resulted in a remarkable increase in *LrP5CDH* expression level in all tissues compared with the control. *LrP5CDH* expression dominated under 250 mM NaCl treatment, but the leaf expression was stable under 50 mM and 250 mM NaCl treatments (Figure 5I).

## 3. Discussion

### 3.1. The Effects of NaCl Treatments on Growth of L. ruthenicum

Xerohalophytes differ in their ability to tolerate salinity due to changes in their morphological, anatomical, and physiological characteristics, which have evolved over a long period of time [35]. *L. ruthenicum* grew well when NaCl concentration was between 50 and 100 mM [36]. In addition, Wang et al. [37] proved that appropriate addition of NaCl can promote the growth of *L. ruthenicum*. These findings were confirmed in our study, in which 50 and 100 mM promoted root length; 100 mM promoted shoot length, root length, and root fresh weight; while 150 induced leaf and stem fresh weight. In other halophytes and xerohalophyte species, such as *Lycium barbarum*, *Sporobolus marginatus*, *Suaeda salsa*, and *Atriplex halimus* [38,39,40], similar results were reported. On the contrary, the application of different NaCl concentrations (25 to 150 mM) to glycophyte species, such as *Zea mays* and *Helianthus annuus*, resulted in a significant reduction in shoot and root length as well as biomass compared to control plants [41]. This confirmed that moderate NaCl treatment enhanced the growth of *L. ruthenicum* [36,42].

The leaves are more sensitive, but also more flexible to environmental stresses. After 14 days of treatment, the osmotic potential in leaves decreased with the increase in external NaCl concentrations. When the osmotic potential is lower, the osmotic adjustment capacity is higher, which makes it easier for plants to absorb water and possibly retain their turgor under low water potential situations. A similar phenomenon was reported in the xerohalophyte species *Atriplex canescens* [43,44]. In general, stomata exposed to higher NaCl treatment closed relatively more than under moderate NaCl treatment. It is well known that stomatal movement and transpiration are related to the water balance in higher plants [45]. Our results showed a decrease in the stomata of *L. ruthenicum* leaves with the increase in external NaCl; this could be a strategy to prevent water loss by transpiration because translocation of water and solutes are disrupted in the presence of osmotic stress, thereby affecting the physiological activity of the plant [46,47]. Accordingly, plants do not only reduce water loss by adjusting the stomatal conductance and transpiration rate, but also enhance water uptake by osmotic adjustment, which can be mediated through accumulation of compatible solutes, such as proline [48,49]. The increase in guard cell size and epidermal cell size in *L. ruthenicum* is hypothesized to increase turgor pressure and water retention capacity, allowing the plant to cope efficiently to high stress through osmotic adjustment.

### 3.2. Effects of NaCl Treatments on Antioxidant Enzyme Activities and Proline Content

The activities of antioxidant enzymes are closely related to the antioxidant defense system and salt tolerance in plants, but these enzyme activities differ among plant species [50]. In this study, increasing NaCl treatments enhanced SOD and CAT activities significantly at 200 and 250 mM NaCl. Valderrama et al. [51] reported a key role for antioxidants under salt stress, when SOD and CAT activities were increased significantly in glycophyte species *Olea europaea* leaves subjected to 200 mM NaCl. Another study also showed that *Myrica rubra* SOD and CAT activities drastically improved when cultivated under oxidative stress [52]. According to Mo et al. [53], the activity of SOD in salt stressed stems of *L. ruthenicum* was significantly higher than the control. Meanwhile, the activity of POD in this study decreased gradually in response to NaCl concentrations, similar phenomenon of POD activity was found in halophyte species *Karelinia caspia* under high salt stress [54]. In three *Malus* species under salt stress, the activity of POD increased on the fourth day after treatment but decreased on eighth and twelfth days [55]; this may suggest that 14 days of NaCl treatment was probability too much for analysis of POD activity in *L. ruthenicum*.

When exposed to salt stress, plants always produce a large amount of reactive oxygen species (ROS), which then cause oxidative stress. In this study, H_2_O_2_ concentration was revealed to be inhibited in a dose-dependent manner among the NaCl concentrations. CAT, SOD, POD, and ascorbate peroxidase (APX) are the main antioxidant enzymes that scavenge excess ROS in plants [56]. SOD is regarded as the key enzyme that facilitates the catalyzing of O_2_^•−^ to H_2_O_2_ whereas CAT rapidly decomposes H_2_O_2_, producing H_2_O and O_2_ [47,54]. In this study, we discovered that the higher the SOD and CAT activities at 200 and 250 mM NaCl, the lower H_2_O_2_ level. Li et al. [57] reported that the exogenous application of H_2_O_2_ to seedlings of glycophyte *Triticum aestivum* under salt stress increased SOD and CAT activities. Thus, our results suggest that the coordination of CAT activity with SOD activity plays a central protective role in the O_2_^•−^ and H_2_O_2_ scavenging process in *L. ruthenicum* explants for better survival under stress. POD is reported to be involved in scavenging of H_2_O_2_, but its role is unclear in *L. ruthenicum* since NaCl treatments inhibited its activity.

Proline content in all tissues was elevated significantly under 250 mM NaCl treatment, with no evidence of explant injury, suggesting that proline may also be involved in physiological response of *L. ruthenicum* to high NaCl concentrations. In xero-halophyte species *A. canescens*, proline accumulation was highly elevated by high salinity (200 and 400 mM NaCl) but was insignificantly affected by moderate concentration (100 mM) of NaCl. Proline can also reduce oxidative damage through stabilization of antioxidant enzymes in stressed plants [20]. This study showed that proline content, SOD and CAT activities increased with increasing NaCl concentrations. Salt stress is reported to inhibit activities of CAT and POD of halophyte species *Pancratium maritimum*; however, their activities were promoted significantly in the presence of exogenous proline [58]. Also, exogenous proline increased CAT activity in glycophyte *O. europaea* leaves under stress [59]. This indicates that a high proline level in *L. ruthenicum* may protect antioxidant enzyme machinery and thus protect the plant from oxidative damage under stress conditions.

The increase in the accumulation of soluble protein and proline indicate their contribution to osmotic adjustment under NaCl treatments. In addition, the contribution of proline to Ψs showed significant increase under high concentration of NaCl treatments and accounted for 1.5% under 250 mM NaCl. Also, in halophytic *Atriplex canescens*, it was found that proline accumulation was significantly correlated to Ψs under high salinity conditions (200 and 400 mM NaCl) [43,44]. This indicates that the addition of NaCl facilitates the accumulation of soluble protein and proline and enhances the function of proline in Ψs to improve NaCl resistance of *L. ruthenicum.*

### 3.3. Proline Metabolism in L. ruthenicum under NaCl Treatments

Since proline accumulation is the result of a combination of biosynthesis and catabolism, it was necessary to analyze the activities of proline enzymes, the genes associated with metabolism, and their relative expression patterns involved in these processes to understand the mechanism of proline metabolism. Proline biosynthesis is a reductive pathway and uses NADPH to reduce glutamate to P5C and P5C to proline and generates NADP^+^ that can be further used as an electron acceptor in the oxidative pentose phosphate pathway [60]. Plants contain P5CS isoenzymes that are capable of catalyzing the first unique reaction of proline synthesis. In the main pathway of proline biosynthesis, Glu is converted to GSA/P5C, which is an intermediate of proline biosynthesis and catabolism [23]. Proline biosynthesis from Glu mainly occurred in the cytosol and chloroplast via P5CS and P5CR. In *L. ruthenicum*, P5CS activity and expression of *LrP5CS1*, *-2* and *-3* genes were promoted under high NaCl treatments. Other scholars have reported that in hybrid glycophyte *Medicago sativa*, increased in P5CS activity and *P5CS* expression after NaCl treatment revealed an involvement of P5CS in proline biosynthesis [56]. Also, in halophyte *K. virginica*, it is reported that overexpression of *KvP5CS1* resulted in proline hyperaccumulation [50], and the overexpression of *VaP5CS* and *LrP5CS* in some glycophyte species such as *Vigna aconitifolia* and *Lilium regale* revealed proline accumulation and consequently enhanced stress tolerance [50,61,62,63]. This suggests that P5CS provides biochemical support for proline biosynthesis in *L. ruthenicum* under high NaCl concentration. In the cytosol, the P5C intermediate is reduced to proline by P5CR. Similar to *L. ruthenicum*, it was confirmed by Armengaud et al. [64] and Verbruggen et al. [65] that P5CR is encoded by only one isoenzyme in most plant species studied. In *L. ruthenicum*, analysis of P5CR activity and *LrP5CR* gene revealed that NaCl treatments elevated its activity and relative expression. Correlation between increasing P5CR activity and proline accumulation was observed in halophytic *Mesembryanthemum crystallinum*, and a moderately salt-tolerant *Morus alba* in NaCl stress [66,67]. Overall, this study confirmed the main proline metabolism pathway (Glu) in *L. ruthenicum* faced with different levels of NaCl treatments, as *LrP5CS1*, *-2*, *-3 and LrP5CR* genes were greatly expressed.

Studies have revealed that the Glu pathway is essential for proline biosynthesis during stress regulation in most species rather than the Orn pathway [68]. δ-OAT has been reported to be involved in the conversion of Orn into GSA and vice versa, using α-ketoglutarate (α-KG) and Glu as co-substrates [23]. In *L. ruthenicum*, two isoforms of δ-OAT were discovered, δ-OAT activity and the relative expression levels *LrOAT1* and *-2* were promoted when faced with NaCl concentrations. Funck, Stadelhofer, and Koch [21] showed localization of GFP-tagged OAT in the mitochondria. There are conflicting hypotheses about the direct role of δ-OAT in proline metabolism, since there is no clear evidence for mitochondrial transport of P5C into the cytosol [23]. Our results revealed that δ-OAT activity and gene expression contributed to proline synthesis in *L. ruthenicum*. However, there is no clear evidence of mitochondrial P5C moving to the cytoplasm or chloroplast for proline synthesis; hence, Glu supplied by δ-OAT is fed into the standard pathway for proline synthesis. More direct studies are required to confirm proline biosynthesis via δ-OAT in *L. ruthenicum*.

The function of proline is dose-dependent, and a high concentration could cause a toxic effect in plants [15,68]. Proline catabolism to Glu occurs in mitochondria via two enzymatic steps: ProDH and P5CDH catalysis. Two genes of ProDH are identified in *L. ruthenicum*; a similar number of genes has also been described in the genomes of *Nicotiana tabacum* cv. [65]. The relative expression of the two genes (*LrProDH1* and *-2*) and ProDH activity analysis revealed an elevated response in NaCl treatments in this study. However, it has been suggested that one *ProDH* gene serves general metabolic functions, whereas the other is involved in stress-induced adaptation mechanisms [65,69]; thus, based on their response in our study, it is suggested that both genes may be responsible for both metabolic enzymes and stress defense responses. Though we did not analyze the activity of P5CDH, NaCl treatments increased transcript level of *LrP5CDH*, which is the second enzyme of proline catabolism.

## 4. Materials and Methods

### 4.1. Plant Material, Growth Condition, and NaCl Treatments

Explants of *L. ruthenicum* used for this study were conserved in Tissue Culture Laboratory of College of Forestry, Gansu Agricultural University, China. About 2–3 cm young shoots were used as explants, and the roots of the explants were induced using ½ Murashige and Skoog nutrient medium (MS medium) supplemented with 0.2 mg/mL indol-3-butyric acid (IBA) and 1mg/mL naphthalene acetic acid (NAA). All cultured explants were incubated in growth chamber at 25/22 °C (day/night) with a relative humidity of 65% and a photoperiod of 16/8 h (light/dark) for three weeks. After rooting, explants of uniform growth (10 cm) were transplanted into rectangular shape plastic vessels with modified ½ Hoagland nutrient solution [70] to develop for 2 weeks before NaCl treatments were introduced. NaCl was supplemented to modified ½ Hoagland nutrient solution to obtained six concentrations of NaCl: 50, 100, 150, 200, 250, and 300 mM. Each treated vessel had 3 replications and 2 explants; therefore, 6 explants per treatment were regarded as multiple replicates. Control explants were maintained in modified ½ Hoagland nutrient solution without NaCl treatment. The nutrient solution for both control and treated containers were changed every 3 days to avert reduction in nutrient availability. The leaves, stems, and roots were collected for measurement of different physiological indexes.

Stress responses involve slower adjustments in enzymatic activity that may not be fully evident in shorter time periods, but transcript levels of genes have been reported to exhibit rapid and transient induction of their expression upon stress exposure, which is important for activation of defense mechanisms and initiation of adaptive processes [71]. Therefore, 14 days were used for enzyme activity and physiological status of *L. ruthenicum* explants, whereas 6 and 12 hours (h) were used for analysis of gene expression in response to NaCl treatment. For gene expression, the explants were exposed to 0, 50, and 250 mM NaCl and placed in a growth chamber at 25 °C and 65% relative humidity using the same experimental design. Under the same light condition, the treatments were applied from 8 a.m. to 8 p.m., and samples were collected at 2 p.m. for 6 h and then at 8 p.m. for 12 h. The leaves, stems, and roots were separated after thorough cleaning with double distilled water and quickly frozen in liquid nitrogen and stored at −80 °C for further analysis. 

### 4.2. Leaf Surface Structural and Osmotic Potential (Ψs) Analysis

The epidermal surface of leaves from 14 d explants under NaCl treatments (0, 50, and 250 mM) was examined with a scanning electron microscope (S-3400N, HITACHI, Shenzhen, China), and 10 mm pieces were cut out of the leaves. The pieces were mounted on SEM stubs with microscopic slide; the slide was cooled with ice close to 0 °C prior to insertion into the SEM in order to slow down desiccation. Four images on both abaxial and adaxial sides of the leaves were taken. ImageJ version 1.47 (National Institutes of Health, Bethesda, MD, USA) was used for determination of the following parameters: stomata density, which was manually counted from all the pictures; stomata size (μm^2^), which is defined as an ellipse with its major axis equal to guard cell length and its minor axis equal to the width of the entire stoma; guard cell size (μm^2^), which is length and width of the guard cell; and leaf epidermal cell area (μm^2^), which was calculated through the number of pixels inside the borderline. 

The leaf Ψs was analyzed using Fiske^®^ Micro-Osmometer Model 210 (Fiske^®^ Associates, Norwood, MA, USA). Fresh leaf tissue of about 0.5 g was collected in a freezing point mode at room temperature, transferred to a microcentrifuge tube, and finely ground using TGrinder (Tiangen Biotech Co., Ltd., Beiing, China). The sample supernatant was collected after centrifugation at 12, 000× *g* for 15 min at room temperature [72]. The van’t Hoff equation was used to calculate Ψs: −n × RT, where n is value of instrument readings, R is the pressure constant (0.008314), and T is the temperature (293 °C) [43,73]. Then, the contribution of proline to leaf Ψs was determined as COP/Ψs × 100%, where COP is the calculated osmotic potential value of proline [73].

### 4.3. Osmolyte Accumulation, Antioxidant Activity, Metabolic Enzyme Content, and H_2_O_2_ Content Determination

The proline accumulation (no. PRO-1-Y) and soluble protein (no. BCAP-2-W), antioxidant enzyme activity (CAT (no. CAT-1-Y), SOD (no. SOD-1-Y) and POD (no. POD-1-Y)), metabolic enzyme activity ((P5CS (no. P5CS-1-Y), P5CR (no. P5CR-2-W), δ-OAT (no. OAT-1-Y), ProDH (no. ProDH-1-Y)), and H_2_O_2_ (no. H_2_O_2_-1-Y) content were determined using assay kits from Comin Biotechnology Co., Ltd., Suzhou, China (www.cominbio.com). All absorbance values were read using Perkin Elmer Singapore Pte. Ltd., Enspire^®^ Multimode plate reader.

Proline and soluble protein content, CAT, SOD, and POD activities were performed according to the Comin Biotechnology Co., Ltd., Suzhou, China, methods described by Wang, Gao, Sun, Lu, Li, Li, Wang, and Liu [55]. All the methods used are previously established techniques that are modified; the acid ninhydrin method was used to determine proline content [74], the bicinchoninic acid (BCA) method [75] was used for soluble protein content, the nitroblue tetrazolium (NBT) method [76] used for SOD activity, the guaiacol method [77] was used to perform POD activity, and the ultraviolet absorption method [78] was employed to determine CAT activity.

H_2_O_2_ was evaluated using a test kit based on the titanium sulfate principle [79] with some similarities as described by Han et al. [80]. H_2_O_2_ and titanium sulfate form a yellow titanium peroxide complex that exhibits characteristic absorbance at 415 nm. Briefly, 0.1 g of fresh sample was homogenized in 1 mL of extraction solution, and the homogenate was centrifuged at 8000× *g* for 10 min at 4 °C. Absorbance values were recorded as Am415 nm for the measuring tube and Ac415 nm as the control tube. Change in A (ΔA) was calculated as A measuring tube—A control tube. H_2_O_2_ content = 1.34 (ΔA − 0.0006)/W, where *W* represents the sample fresh weight. 

P5CS activity was assayed based on the glutamyl phosphate reductase activity as described previously by Campanile et al. [81] with some modifications. Briefly, 0.1 g of fresh sample was homogenized in 1 mL of extract solution (Tris-HCl buffer containing MgCl_2_∙6H_2_O, KCl, EDTA, DTT, and PVP) and centrifuged at 8000× *g* for 10 min at 4 °C. The supernatant was collected for enzymatic reaction. The enzymatic reaction consisted of 100 µL supernatant and 100 µL Tris-HCl buffer (containing glutamic acid, ATP, hydroxylamine hydrochloride, and MgCl_2_∙6H_2_O); then, the reaction was placed under 37 °C for 10 min and centrifuged at 8000× *g* for 10 min at 25 °C. The supernatant was collected for chromogenic reaction, which contained 20 µL of supernatant and 200 µL chromogenic mixture (H_2_O, ASA, ammonium molybdate, and H_2_SO_4_ in a 2:1:1:1 ratio) (test sample). The reaction was then placed under room temperature for 30 min, and the absorbance was recorded at 660 nm. A blank control, standard control and positive control were also measured, which contained 20 µL of sterilized water, 20 µL of standard phosphorus application solution, and 20 µL of enzymatic control. The control tube for the enzymatic reaction was not incubated for 10 min. This method has the characteristics of traceability, sensitivity, and rapidity; therefore, the test tubes used in the determination are strictly required to be phosphorus-free. The P5CS activity (U/mg protein) was calculated as standard control × (assay sample − positive control) ÷ (standard control − blank control) × total volume of enzymatic reaction ÷ (sample protein content × volume of extract used) ÷ reaction time.

The extraction and analysis of P5CR activity was based on the method of Rena et al. [82]. Briefly, fresh sample was homogenized in 1 mL of extract solution (Tris-HCl with NaCl) and then centrifuged at 12,000× *g* for 10 min at 4 °C. The supernatant was collected for the final reaction. The final reaction mixture contained 10 µL of supernatant and 190 µL of working solution (90 µL NAD, 90 µL L-thiazolidine-4-carboxylic acid, 10 µL of solution 4 (WST-8, 1-mPMS, and NaCl)). The control reaction mixture contained 10 µL supernatant and 190 µL control solution (90 µL Tris-HCl, 90 µL NAD, and 10 µL of solution 4). The reactions were incubated at 37 °C in dark for 30 min, and the absorbance was recorded at 450 nm. Change in absorbance (ΔA) was calculated as final reaction − control reaction, and the P5CR activity was calculated as (△A + 0.0285) ÷ 1.3384 × total volume of enzymatic reaction ÷ (sample protein content × volume of extract used) × 1000 ÷ reaction time.

δ-OAT activity analysis was performed according to Charest et al. [83] with some modifications. A quantity of 0.1 g fresh sample was homogenized in 1 mL of extraction solution (K_2_HPO_4_∙H_2_O-KH_2_PO_4_ buffer solution containing EDTA, mercaptoethanol, and glycerol) and then centrifuged at 10,000× *g* for 10 min at 4 °C, and the supernatant was collected for final reaction. The final reaction mixture contained 20 µL supernatant, 60 µL ornithine, 60 µL α-ketoglutaric acid, and 60 µL NADH, and the change in absorbance was calculated at 340 nm from initial absorbance and final absorbance at 37 °C for 10 min reaction. The activity of δ-OAT was calculated according to the sample protein content. 

ProDH activity was measured using methyl isothiocyanate. Briefly, 0.1 g of fresh sample was homogenized in 1 mL of extract solution (Na_2_HPO_4_-NaH_2_PO_4_ buffer solution with EDTA). After centrifugation at 1500× *g* for 15 min at 4 °C, 10 µL of TritonX-100 was added to the supernatant and placed in an ice bath for 30 min, then centrifuged at 16,000× *g* for 20 min at 4 °C. The supernatant was collected for final reaction, which contained 35 µL supernatant, 15 µL PMS, and 150 µL mixed solution (2.4 mL Na_2_CO_3_-NaHCO_3_ buffer solution: 0.3 mL L-proline: 0.3 mL DCPIP), and the decrease in absorbance was monitored at 600 nm in an interval of 10 min. One enzyme activity unit was defined as the alternation of 0.01 absorbance in 1 mL of reaction system per minute by 1 mg of protein, and the result was expressed as U/mg protein.

### 4.4. Bioinformatic Analysis of Proline Metabolic Enzyme Genes

The sequence information of proline metabolic enzymes P5CS, P5CR, δ-OAT, ProDH, and P5CDH was obtained from Sequence Read Achieve (SRA) database of NCBI (SRR7700825) [33] and our Supplementary data file S2 and S3. The sequences were analyzed using DNAMAN version 8 software, confirmed by online BLAST search on the NCBI database and HMMER software version 3.3.2 [84] was used to additional authenticate the putative proteins data with *A. thaliana* as query with E-value set to 1 × 10^−10^. ProtParam online software (https://web.expasy.org/protparam/, accessed on 23 September 2022) was used to analyze the basic characteristics of the encoded proteins. Protein subcellular localization prediction was performed using the online DeepLoc-1.0 (https://services.healthtech.dtu.dk/services/DeepLoc-1.0/, accessed on 23 September 2022) tool.

### 4.5. RNA Extraction, cDNA Synthesis, and RT-qPCR Analysis

Total RNA was extracted from leaf, stem, and root using TransZol Up Plus RNA Kit (no.M31018) (TransGen Biotech Co., Ltd., Beijing, China) conferring to the manufacturer’s instruction. The RNA quantity and quality were determined using TGen Spectrophotometer (Tiangen Biotech Co., Ltd., Beiing, China) based on the A260 nm/A280 nm and A260 nm/A230 nm ratios. *Evo* M-MLV RT Kit, no. AG11705 (Accurate Biotechnology Co., Ltd., Hunan, China) was used to reverse-transcribe the total RNA into cDNA and for removal of any possible genomic DNA mixed in the cDNA before RT-qPCR analysis, following the manufacturer’s instruction.

The RT-qPCR analysis was conducted following the minimum information for publication of quantitative real-time PCR experiments (MIQE) guidelines [85] and gene primers were designed using Primer premier version 5.0 software and synthesized by TsingKe Biological Technology Co., Ltd., Xi’an, China (Table 4). The *L. ruthenicum Elongation factor1-*α (*LrEF1-α*) gene (JX427553) was used as a housekeeping gene (https://ngdc.cncb.ac.cn/icg/search?term=Lycium, accessed on 23 September 2022). Synthetic cDNA was diluted in a 4-fold series (1, 10, 100, and 1000) and the method described by Chang et al. [86] was used for primer evaluation and standard curves analysis. Also, the primer pair specificity was confirmed using single peak melting curve analysis (Supplementary Figures S1 and S2). Using Heiff^®^ qPCR SYBR Green Master Mix kit (Yeasen Biotech Co., Ltd., Shanghai, China), three biological replicates and triplicate quantitative analysis were performed according to the manufacturer’s protocol. The RT-qPCR assay was carried out using the QuantStudio™ 5 Real-Time PCR Instrument (ABI) (Life Technologies Holdings Pte. Ltd., Singapore). The 2^−ΔΔCt^ method [87] was used to determine target gene expression changes based on normalization with the reference gene, and 0 Na—6 h leaf was used as the reference condition.

### 4.6. Statistical Analysis

Data of the three biological replicates tooling six explants per treatment and 3 analytical replicate were performed using a general linear model (SPSS version 23); the means and standard deviation (SD) of replicates were calculated. Data were compared between treatments and control by analysis of variance (ANOVA) in SPSS version 23 to separate significant differences, followed by Duncan’s multiple range tests with least significant difference (LSD) at *p* ≤ 0.05. ANOVA was used to analyze the differences between treatments in each tissue and time. All graphical presentations were performed using Graphpad Prism version 8.

## 5. Conclusions

In this study, it was shown that *L. ruthenicum* can resist high NaCl concentration, and moderate NaCl concentration is useful for growth promotion and biomass production. Increasing NaCl concentration increased the contents of SOD and CAT, but inhibited the activity of POD, which enhanced the antioxidant system of *L. ruthenicum* to scavenge ROS. Our results revealed that NaCl treatment leads to an increase in proline level in *L. ruthenicum*, which suggested proline protection of antioxidant enzyme machinery and thus protected the plants from oxidative damage under NaCl treatment. The increase in P5CS, P5CR, and δ-OAT activities in response to NaCl treatments promoted the level of proline in *L. ruthenicum*. Proline metabolism enzymes related genes *LrP5CS1*, *-2*, *-3*, *LrP5CR*, *LrOAT-1*, *-2*, *LrProDH-1*, *-2*, and *LrP5CDH* were identified. RT-qPCR analysis confirmed that their expression profiles were promoted in response to NaCl treatments, indicating their roles in proline metabolism. Overall, the results of this study confirmed that the Glu pathway is involved in proline accumulation in *L. ruthenicum* under stress conditions.

## Figures and Tables

**Table 1:** Phenotypic traits and biomass response of *L. ruthenicum* to NaCl treatments.

NaCl (mM)	Shoot Length (cm)	Root Length (cm)	Leaf Fresh Weight (g)	Stem Fresh Weight (g)	Root Fresh Weight (g)
0	19.14 ± 0.18 ^b^	18.72 ± 0.37 ^b^	2.03 ± 0.23 ^c^	0.69 ± 0.01 ^c^	1.11 ± 0.07 ^b^
50	19.22 ± 0.14 ^ab^	19.84 ± 0.24 ^a^	2.59 ± 0.18 ^b^	0.74 ± 0.03 ^bc^	1.18 ± 0.12 ^b^
100	19.77 ± 0.19 ^a^	19.56 ± 0.30 ^a^	2.69 ± 0.09 ^b^	0.78 ± 0.01 ^ab^	1.45 ± 0.05 ^a^
150	19.15 ± 0.32 ^b^	17.02 ± 0.26 ^c^	3.34 ± 0.15 ^a^	0.82 ± 0.04 ^a^	1.04 ± 0.14 ^c^
200	18.42 ± 0.15 ^c^	16.40 ± 0.10 ^cd^	1.66 ± 0.11 ^cd^	0.36 ± 0.02 ^d^	0.43 ± 0.10 ^cd^
250	16.16 ± 0.19 ^d^	15.74 ± 0.20 ^d^	1.35 ± 0.13 ^de^	0.15 ± 0.02 ^e^	0.20 ± 0.06 ^d^
300	14.24 ± 0.25 ^e^	14.38 ± 0.11 ^e^	1.05 ± 0.11 ^e^	0.13 ± 0.01 ^e^	0.13 ± 0.04 ^d^

Values are the means ± SD. Different subscript letters following values in the same row show significant differences among different NaCl treatments using Duncan’s multiple range test at *p ≤* 0.05.

**Table 2:** The average parameters of stomata size, guard cell size, and epidermal cell size.

NaCl (mM)	Stomata Density		Stomata Size (μm^2^)		Guard Cell Size (μm^2^)		Epidermal Cell Size (μm^2^)	
	Adax	Abax	Adax	Abax	Adax	Abax	Adax	Abax
0	39	33	26.93 ± 1.9 ^b^	28.48 ± 3.8 ^b^	36.64 ± 1.0 ^bc^	40.92 ± 0.8 ^b^	19,600 ± 469 ^c^	21,620 ± 738 ^c^
50	27	21	42.68 ± 3.5 ^a^	38.73 ± 1.9 ^a^	39.48 ± 0.7 ^b^	35.26 ± 1.6 ^bc^	25,716 ± 3169 ^c^	33,550 ± 735 ^b^
250	20	14	3.93 ± 0.6 ^c^	2.63 ± 0.3 ^c^	60.98 ± 3.9 ^a^	32.13 ± 2.4 ^c^	36,794 ± 3565 ^b^	52,322 ± 3105 ^a^

Values are the means ± SD in the samples. Different subscript letters following values show significant differences among different treatments and leaf surface using Duncan’s multiple range test at *p* ≤ 0.05.

**Table 3:** The basic features of the identified sequences of proline metabolic proteins.

Protein Name	Protein ID	Aa Residues	MW (kDa)	pI	Subcellular Localization Prediction
LrP5CS1	TRINITY_DN21284_c1_g3_i9_3	387	41.94	5.72	cyto
LrP5CS2	TRINITY_DN21540_c0_g2_i1_2	717	77.59	6.11	cyto
LrP5CS3	TRINITY_DN25963_c0_g2_i9_10	588	63.52	5.55	cyto
LrP5CR	TRINITY_DN15477_c0_g1_i2_5	274	28.44	8.92	cyto
LrOAT1	TRINITY_DN11125_c0_g1_i4_8	320	35.11	8.53	mito
LrOAT2	TRINITY_DN13448_c0_g1_i1_6	319	35.02	8.53	mito
LrProDH1	TRINITY_DN36679_c0_g1_i1_7	501	56.20	8.24	mito
LrProDH2	TRINITY_DN50_c0_g1_i2_8	489	54.92	7.97	mito
LrP5CDH	TRINITY_DN23202_c0_g1_i3_9	448	50.15	6.38	mito

Aa: Amino acid residues; MW: molecular weight; pI: isoelectric point; mito: mitochondrion; cyto: cytoplasm.

**Table 4:** Primers information for proline metabolic enzymes genes.

Gene	Primer Name	Sequence (5′–3′)	E(%)	R^2^
*LrP5CS1*	*LrP5CS1-F*	CATCATCTCCATTGGGTGTTCT	99	0.99
	*LrP5CS1-R*	CCTCCTTCCCTCCTTTCAGC		
*LrP5CS2*	*LrP5CS2-F*	GCGGAGGTGGGCATTAGTA	93	0.99
	*LrP5CS2-R*	CTTGCGAGCCATTTAGTTGTTA		
*LrP5CS3*	*LrP5CS3-F*	CATTGGCCGCATTTTAAAGAG	105	0.99
	*LrP5CS3-R*	TGTACGAGTGCATCAGGTCGTG		
*LrP5CR*	*LrP5CR-F*	CACCAGGGGGAACAACCATT	99	0.99
	*LrP5CR-R*	TGCGTTTAGCTGCACCAACAA		
*LrOAT1*	*LrOAT1-F*	TCTTCCCCTTCTCAAAATCTCA	100	0.99
	*LrOAT1-R*	CCCAGACGGATGAACCCTTAG		
*LrOAT2*	*LrOAT2-F*	GTCAGCATTGATGTCCAGTTTCT	107	0.99
	*LrOAT2-R*	TGTCCACATTGCTAGCTGGT		
*LrProDH1*	*LrProDH1-F*	ATTCAGGGAGGTAATAATGGGG	103	0.99
	*LrProDH1-R*	GTAACCGTCCTTCGTACTTCTTCT		
*LrProDH2*	*LrProDH2-F*	CTCTAACGGCAGAGGAAGAGC	101	0.99
	*LrProDH2-R*	CCGCATCAATGAGTAAGGGA		
*LrP5CDH*	*LrP5CDH-F*	TGCCGTCAATCATGTATCG	100	0.99
	*LrP5CDH-R*	ATTGTGGTTTGACCTTGC		
*LrEF1-*α	*LrEF1a-F*	CCATACCAGCATCACCATTCTTC	100	0.99
	*LrEF1a-R*	GTCACACTTCCCACATTGCC		

## Data Availability

The data are contained within this article.

## Supplementary Materials

The following supporting information can be downloaded at: https://www.mdpi.com/article/10.3390/ijms241813794/s1.
