# Supplementary material for: Proline Metabolism Process and Antioxidant Potential of Lycium ruthenicum Murr. in Response to NaCl Treatments
Source: Int J Mol Sci. 2023 Sep 7;24(18):13794. doi: 10.3390/ijms241813794 (PMC10530678; doi:10.3390/ijms241813794)
Supplement: Supplementary file 1 [file ijms-24-13794-s001.zip › supplementary figures.pdf]

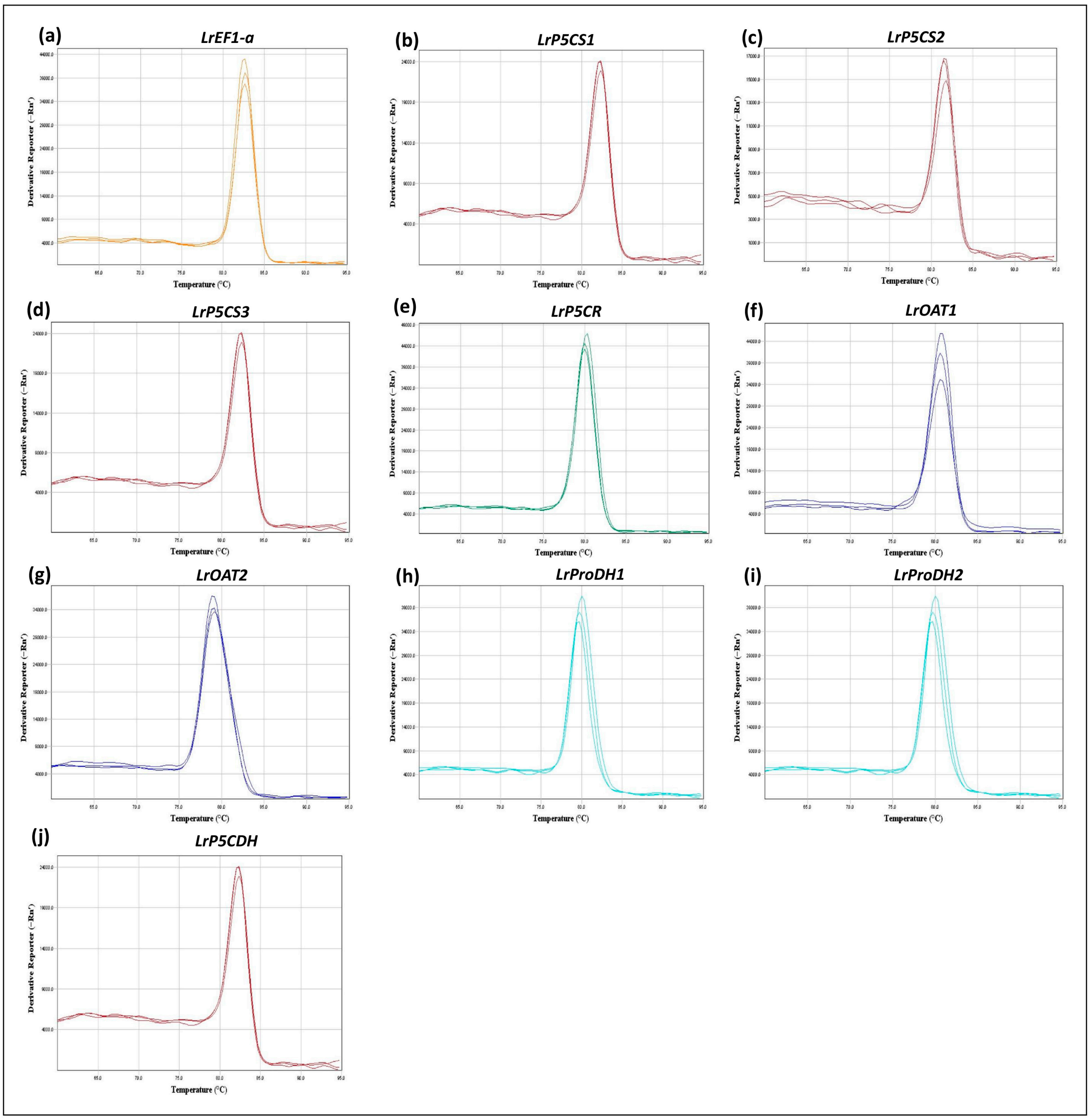

Supplementary figure S1: Validation of primer pairs using qPCR single peak analysis. (a). *LrEF1-α* (b). *LrP5CS1* (c). *LrP5CS2*, (d). *LrP5CS3*, (e). *LrP5CR*, (f). *LrOAT1*, (g). *LrOAT2*, (h). *LrProDH1*, (i). *LrProDH2*, and (j). *LrP5CDH*.

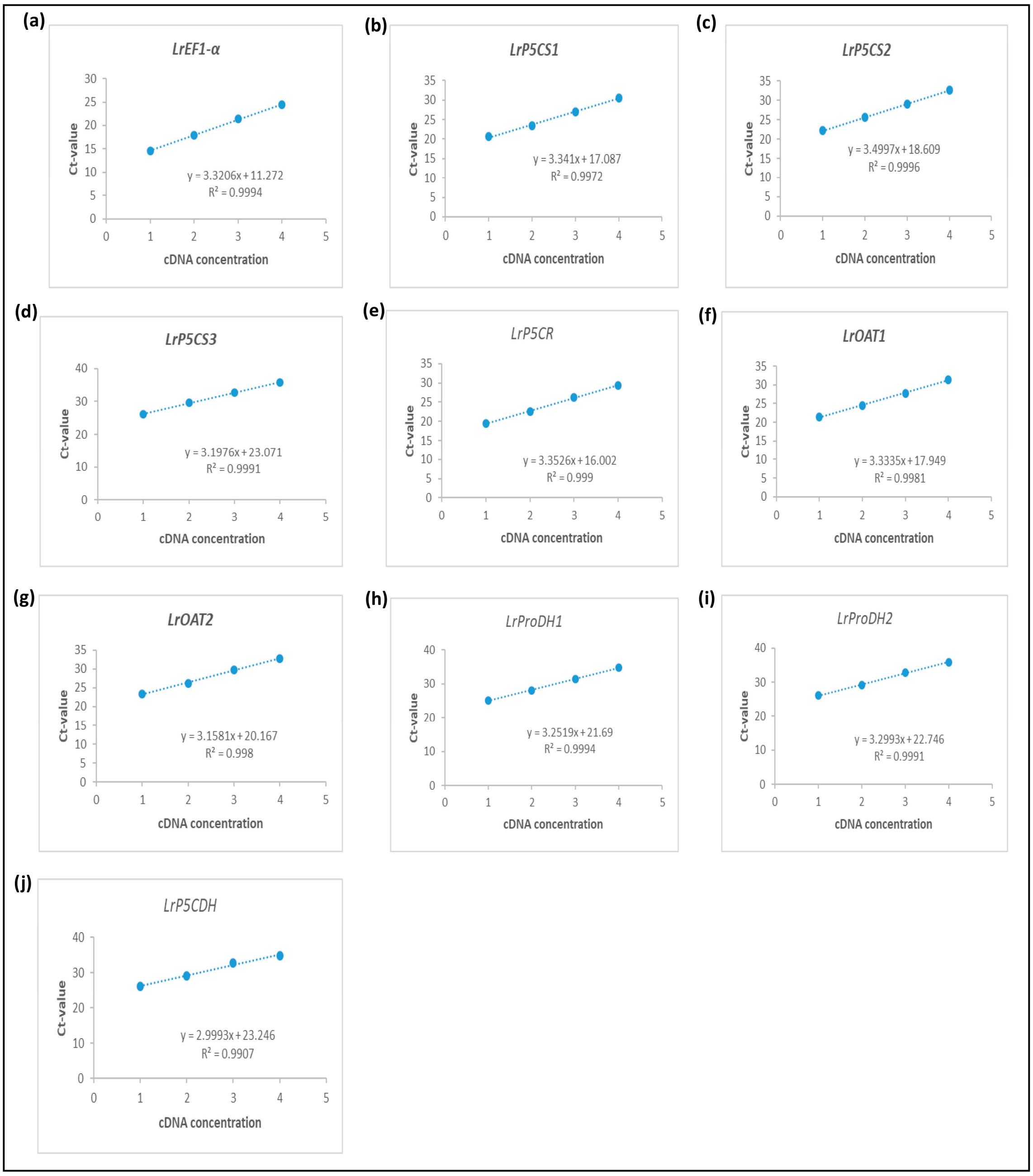

Supplementary figure 2: Standard curve of primer pairs efficiency analysis. (a). *LrEF1-α* (b). *LrP5CS1* (c). *LrP5CS2*, (d). *LrP5CS3*, (e). *LrP5CR*, (f). *LrOAT1*, (g). *LrOAT2*, (h). *LrProDH1*, (i). *LrProDH2* and (j). *LrP5CDH*.

Supplementary Data File S1: Selected candidate metabolic enzyme genes sequence used for this study. Supplementary Data File S2: Assembled Nucleotide Sequences of *L. ruthenicum*. Supplementary Data File S3: Assembled Protein Sequences of *L. ruthenicum*.
